# Accumulation and distribution of mercury in fruiting bodies by fungus *Suillus luteus* foraged in Poland, Belarus and Sweden

**DOI:** 10.1007/s11356-015-5513-4

**Published:** 2015-10-07

**Authors:** Martyna Saba, Jerzy Falandysz, Innocent C. Nnorom

**Affiliations:** Laboratory of Environmental Chemistry and Ecotoxicology, Gdańsk University, 63 Wita Stwosza Str., 80-308 Gdańsk, Poland; Abia State University, Uturu, Abia Nigeria

**Keywords:** Fungi, Mercury, Bioavailability, Bioconcentration, Mushrooms, Wild food

## Abstract

Presented in this paper is result of the study of the bioconcentration potential of mercury (Hg) by *Suillus luteus* mushroom collected from regions within Central, Eastern, and Northern regions of Europe. As determined by cold-vapor atomic absorption spectroscopy, the Hg content varied from 0.13 ± 0.05 to 0.33 ± 0.13 mg kg^−1^ dry matter for caps and from 0.038 ± 0.014 to 0.095 ± 0.038 mg kg^−1^ dry matter in stems. The Hg content of the soil substratum (0–10 cm layer) underneath the fruiting bodies showed generally low Hg concentrations that varied widely ranging from 0.0030 to 0.15 mg kg^−1^ dry matter with mean values varying from 0.0078 ± 0.0035 to 0.053 ± 0.025 mg kg^−1^ dry matter, which is below typical content in the Earth crust. The caps were observed to be on the richer in Hg than the stems at ratio between 1.8 ± 0.4 and 5.3 ± 2.6. The *S. luteus* mushroom showed moderate ability to accumulate Hg with bioconcentration factor (BCF) values ranging from 3.6 ± 1.3 to 42 ± 18. The consumption of fresh *S. luteus* mushroom in quantities up to 300 g week^−1^ (assuming no Hg ingestion from other foods) from background areas in the Central, Eastern, and Northern part of Europe will not result in the intake of Hg exceeds the provisional weekly tolerance limit (PTWI) of 0.004 mg kg^−1^ body mass.

## Introduction

Mercury (Hg) content in the Earth’s crust is considered at 0.08 mg kg^−1^ dry matter (dm) on the average (Rytuba 2003) and in forest topsoil in Poland is less than 0.08 mg kg^−1^ dm. In Poland, with a mosaic-like pattern of soil type, Hg in topsoil (0–10 cm layer) is, on the average, below 0.05 mg kg^−1^ dm (Falandysz et al. 2003). In mercuriferous belt regions, both rock and soil contain elevated concentrations of this element (Fan 1991; Gustin et al. 1999). For example, in randomly collected samples of forest topsoil from the mercury belt region in Yunnan Province of China, the content of Hg was up to 3.4 mg kg^−1^ dm (Kojta et al. 2015). Mercury at greater concentration is found in soils in the cinnabar (HgS) mining sites and sites with cinnabar mine wastes that are sometimes used for crops cultivation and where it can reach concentration, e.g., 120 mg kg^−1^ dm (Qiu et al. 2012), and Hg at such sites can be accumulated by some mushrooms to extraordinary great concentration (Árvay et al. 2014).

Mercury is a toxic metallic element that is liquid in room temperature and which easy evaporates into the atmosphere from the varying sources, both natural and anthropogenic. The natural sources of emission of Hg into ambient air and its environmental release are volcanic eruptions and volatilization and re-vitalization from the earth surfaces. The anthropogenic sources are many because a wide manufacture of Hg in the past from the primarily ore (cinnabar, HgS) together with numerous appliances of Hg and its compounds (UNEP 2013). A legal production of Hg from cinnabar is continuing somewhere as well as illegal artisanal manufacture (Qiu et al. 2012). Another possibility is manufacture as by-product in gold and silver production and from sulfide deposits (Rytuba 2003). Many major uses of Hg declined and others legal are also declining in recent years (UNEP 2013), while not declined amounts emitted annually into the atmosphere (Liang et al. 2015; Zhang et al. 2015). The thermal reactions in process such as burning, combustion, incineration, or smelting lead to voltalization and release into the atmosphere of Hg contained in organic and mineral substrates. The coal and other fuel combustion because of a huge scale of processes are nowadays significant anthropogenic source of Hg and less is a plant biomass burning (Liang et al. 2015; UNEP 2013). A small-scale and artisanal gold production is a leading cause of pollution with Hg of aquatic ecosystems at the local and regional scale and Hg release into the atmosphere in many regions of the world (Olivero-Verbel et al. 2002, 2015; UNEP 2013).

In the atmosphere, Hg slowly undergoes oxidation and is re-deposited and adsorbed on and into topsoil and on other surfaces and this could result from regional or long-range global atmospheric transport, and because of that and continuous emissions from around 200 years, the levels can be elevated in forest topsoil in fragile regions (Falandysz et al. 2014; Nygård et al. 2012; Richardson et al. 2013). Airborne Hg deposited on land surfaces is well retained by upper layer of forest soil, which is particularly rich in organic matter (Demers et al. 2007; Suchara and Sucharová 2002). In industrial areas and especially at sites of nonferrous metal ore smelting or chemicals synthesis with Hg compounds used as catalyst, the Hg content of topsoil is usually elevated and in Poland, the tolerance limit for industrial sites is 30 mg kg^−1^ dry soil (Polish Governmental Gazette 2002).

Surface soil and litter of forests on which mushrooms grow have been reported to contain elevated levels of anthropogenic Hg (Demers et al. 2007). The mushroom mycelia are effective in mobilizing Hg from the substrate (soil, litter, wooden substrate) and translocation and sequestering Hg into the mushroom fruiting bodies though the ability to achieve this and the efficiency vary among species (Melgar et al. 2009; Nasr and Arp 2011; Rieder et al. 2011). Several studies have shown fruiting bodies of some wild-grown mushroom species from the genus such as *Agaricus*, *Boletus*, *Leccinum*, and *Macrolepiota* emerged from the soils of various types and localizations to be much more abundant in Hg compared to some other mushroom genus (Brzostowski et al. 2011a; Chojnacka et al. 2012; Chudzyński et al. 2009, 2011; Drewnowska et al. 2012, 2014; Falandysz and Brzostowski 2007; Falandysz et al. 2002, 2007a, b, 2015; Gucia et al. 2012b; Krasińska and Falandysz 2015a, b; Melgar et al. 2009; Nasr et al. 2012; Seeger and Nützel 1976). Also, rhizomorphs (root-like structures) that are developed by certain mushrooms seem, apart from mycelia, to play a role in Hg absorption from soil solution and transferring it into flesh of fruiting bodies in the case of *Armillaria solidipes* Peck 1900 (Falandysz et al. 2013). Considering the hazardous and toxic nature of Hg and the increasing global emissions and environmental contamination with Hg (UNEP 2013), it becomes important to stop this process and to monitor the levels in environmental flora and fauna, especially well-enjoyed food such as mushrooms for which some species still have dearth of data on their chemical composition.

It is also a common knowledge and tradition that mushrooms are nutritional and that certain mushroom species have medicinal properties. However, available data is not yet comprehensive especially on the chemical composition of wild-grown mushrooms and likely health benefits and risks to consumers. Both the organic and inorganic mercury contents of mushroom of even the same species could vary significantly depending on the place (site) of collection. Preservation method (drying, consumption fresh, etc.) and the cooking or processing methods adopted are also contributory in determining the content, availability/accessibility of these constituents of the mushroom in the ready-to-eat mushroom meal (Falandysz and Drewnowska 2015).

This study is a part of an ongoing wild-grown mushrooms research aimed at investigating the level of Hg contamination and bioconcentration by *Suillus luteus* (L.) Roussel 1796 from forested areas of Poland, Belarus, and Sweden in Central, Eastern, and Northern regions of Europe. Mercury intakes from the consumption of this mushroom will also be estimated and will be compared to established regulatory limits such as the provisional weekly tolerance limit (PTWI) and the RDI. Results of this study will contribute to data necessary in evaluating the nutritional benefits and likely toxicological risks of the consumption of *S. luteus*.

## Materials and methods

**Fig. 1:**
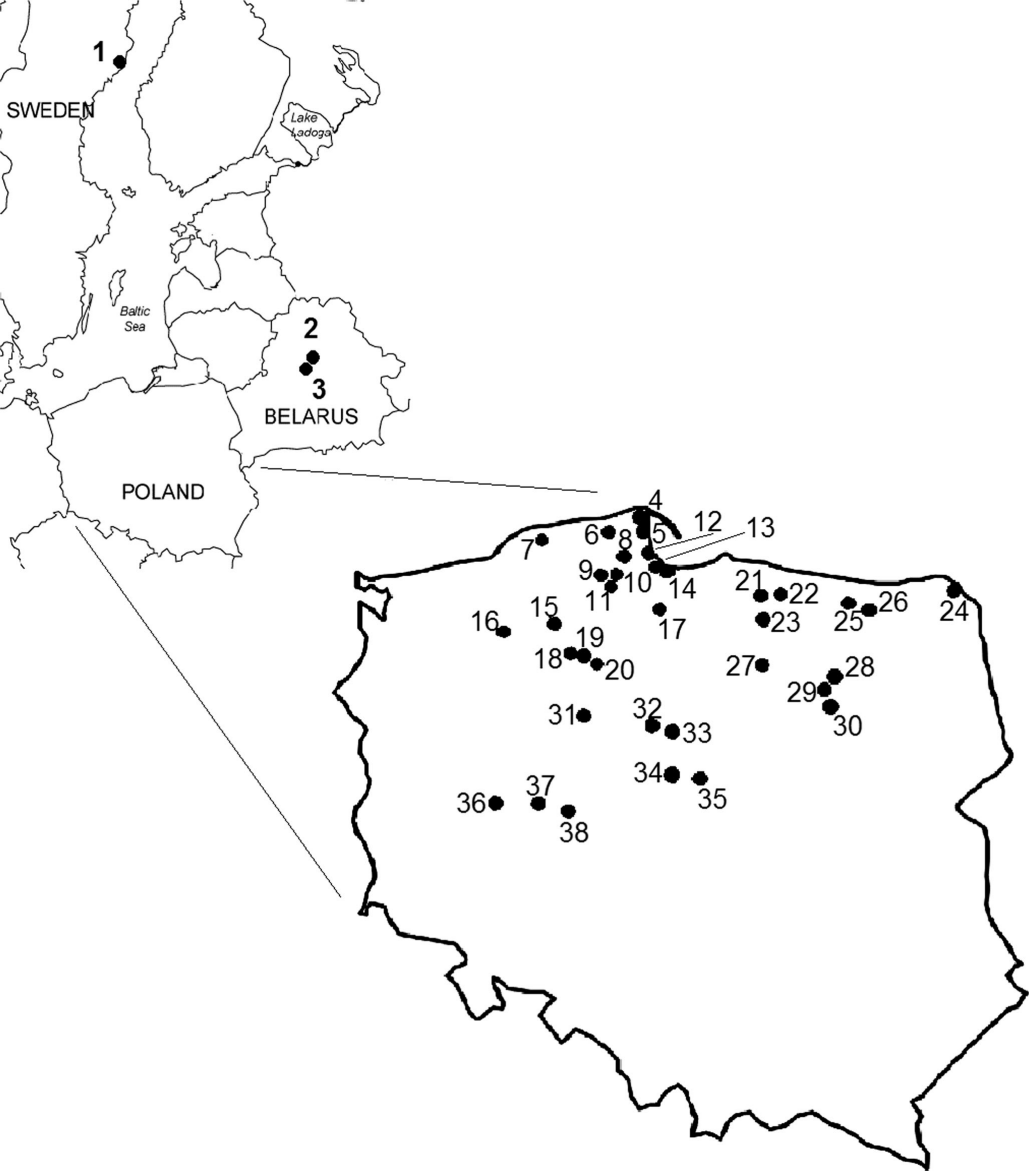
Localization of the sampling sites of mushrooms/soil (*1* Sweden: Umeå, *2* Belarus: Borysów; *3* Belarus: Kostryca, *4* Poland: Darżlubska Wilderness – Ostrowo, *5* Poland: Seaside Landscape Park, *6* Poland: Strzebielino, *7* Poland: Słupsk region, *8* Poland: Darżlubska Wilderness – Strzępcz, *9* Poland: Parchowo, *10* Poland: Stężyca, *11* Poland: Gołubie, *12* Poland: Trójmiejski Landscape Park, *13* Poland: Otomin, *14* Poland: Sobieszewo Island, *15* Poland: Zaborski Landscape Park, *16* Poland: Szczecinek, *17* Poland: Tuchola Pinewoods – Osiek, *18* Poland: Rzecznica, *19* Poland: Tuchola Pinewoods – Śliwice, *20* Poland: Tuchola Pinewoods – Śliwice, *21* Poland: Kaszuny, *22* Poland: Kiwity, *23* Poland: Wysokie, *24* Poland: Augustów Primeval Forest, *25* Poland: Kętrzyn (outskirts), *26* Poland: Giżycko (outskirts), *27* Poland: Olsztynek (outskirts), *28* Poland: Szczytno (outskirts), *29* Poland: Puchałowo, *30* Poland: Mazowsze, Gmina Lipowiec, *31* Poland: Bydgoszcz forests - Białe Błota, *32* Poland: Ciechocinek, *33* Poland: Włocławek forests, *34* Poland: Lubraniec, *35* Poland: Mursk, Goreń, Kukawy, *36* Poland: Porażyn, *37* Poland: Rogalin, *38* Poland: Anastarzewo near Konin)

One thousand seven hundred thirty-eight individual fruiting bodies of *S. luteus* mushroom were picked up from 38 spatially distant sites in Poland, 2 sites in Belarus, and 1 site in Sweden during the mushroom collection season in 1995–2013 (Fig. 1; Tables 1 and 2). On collecting the mushroom fruiting bodies, the topsoil layer of the forests (0–10 cm) beneath the fruiting bodies were also collected for most of the sites studied.

**Table 1:** Mercury content (mg kg^-1^ dry matter) in mushroom *Suillus luteus* and soil substratum from the region of Europe (Belarus, Poland, Sweden), quotient of Hg content in cap to stem (Q_C/S_) and quotient of Hg content in cap/stem to soil substratum beneath the fruiting bodies (BCF, bioconcentration factor); arithmetical mean value ± S.D., median value and range)

Place, year, and sample size		Hg			Q_C/S_	BCF	
		Whole fruiting body					
		Cap	Stem	Soil		Cap	Stem
1ψ	Sweden, Umeå, 1995 *n* = 14*	0.17 ± 0.07	0.074 ± 0.014	0.049 ± 0.041	2.1 ± 0.8	4.6 ± 2.0	2.5 ± 1.3
		0.15	0.073	0.029	1.9	5.1	2.8
		0.075–0.34	0.054–0.097	0.019–0.15	1.1–3.4	1.3–6.7	0.39–4.2
2	Belarus, Borysów, 2012 *n* = 1 (15)	0.15	0.073	NA	2.1	NA	NA
3	Belarus, Kostryca, 2013 *n* = 1 (15)	0.090	0.038	0.026	2.4	3.5	1.5
5	Poland, Seaside Landscape Park, 2006 *n* = 15 (103)	0.16 ± 0.06	0.095 ± 0.038	0.018 ± 0.008	2.0 ± .9	11 ± 7	6.5 ± 5.2
		0.15	0.082	0.018	1.0	8.5	4.5
		0.10–0.34	0.040–0.16	0.0058–0.043	0.71–3.4	2.4–27	1.9–20
8	Poland, Darżlubska Wilderness - Strzępcz, 2003 *n* = 1^#^(78)**	0.18	0.091	0.021	2.0	8.6	4.3
9	Poland, Parchowo, 2010 *n* = 1 (10)	0.15	0.070	0.043	2.1	3.5	3.5
10	Poland, Stężyca, 2000 *n* = 1 (48)	0.33	0.096	0.028	3.4	12	3.4
11	Poland, Gołubie, 2000 *n* = 16 (64)	0.30 ± 0.12	0.076 ± 0.025	0.027 ± 0.017	4.3 ± 1.9	14 ± 9	3.6 ± 1.3
		0.27	0.073	0.023	3.7	11	3.4
		0.16–0.69	0.039–0.16	0.011–0.073	2.0–9.6	2.5–40	0.83–5.7
13	Poland, Otomin, 2002 *n* = 16 (80)	0.19 ± 0.06	0.066 ± 0.028	0.025 ± 0.001	3.1 ± 0.9	7.6 ± 2.6	2.6 ± 1.1
		0.19	0.065	0.025	2.9	7.5	2.5
		0.075–0.30	0.022–0.12	0.023–0.027	2.1–6.1	2.9–12	0.93–4.7
16	Poland, Szczecinek, 2003 *n* = 13	0.30 ± 0.17	0.038 ± 0.014	0.0078 ± 0.0035	5.3 ± 1.3	42 ± 18	6.7 ± 3.2
		0.22	0.035	0.0072	5.5	39	5.9
		0.15–0.61	0.025–0.058	0.0030–0.014	3.6–6.4	18–75	38–11
17	Poland, Tuchola Pinewoods - Osiek, 2001 *n* = 1 (109)	0.17	0.018	NA	9.4		
18	Poland, Rzecznica, 2003 *n* = 1 (109)	0.15	0.016	NA	9.4		
19	Poland, Tuchola Pinewoods – Śliwice, 2000 *n* = 15	0.11 ± 0.03 0.10 0.075–0.16	NA	NA	NA		
20	Poland, Tuchola Pinewoods – Śliwice, 2010 n = 1 (15)	0.13	0.044	0.011	2.5	12	4.0
21	Poland, Kaszuny, 2005 *n* = 1 (9)	0.86	0.67	NA	1.3		
22	Poland, Kiwity, 2002 *n* = 1 (73)	0.20	0.021	NA	9.5		
23	Poland, Wysokie, 2006 *n* = 1 (68)	0.40	0.18	NA	2.2	NA	NA
24	Poland, Augustów Primeval Forest, 2006 *n* = 13	0.13 ± 0.05	0.040 ± 0.012	0.035 ± 0.005	3.3 ± 0.8	3.6 ± 1.3	1.2 ± 0.4
		0.11	0.040	0.033	3.0	3.4	1.2
		0.070–0.23	0.023–0.060	0.030–0.047	2.1–4.9	1.8–6.2	0.64–2.0
30	Poland, Mazowsze, Gmina Lipowiec, 2006 *n* = 1 (27)	0.17	0.017	NA	10		
31	Poland, Bydgoszcz forests - Białe Błota, 2000 *n* = 1 (75)	0.13	0.060	0.014	2.2	9.3	4.3
32	Poland, Ciechocinek, 2004 *n* = 15 (90)	0.16 ± 0.04	0.092 ± 0.019	0.012 ± .002	1.8 ± 0.4	14 ± 5	8.0 ± 2.0
		0.17	0.097	0.012	1.8	13	8.7
		0.10–0.23	0.061–0.13	0.0091–0.017	1.1–2.3	8.0–23	3.7–10
33	Poland, Włocławek forests, 2006 *n* = 15 (60)	0.14 ± 0.04	0.053 ± 0.014	0.053 ± 0.025	3.0 ± 1.6	4.3 ± 4.6	1.4 ± 1.1
		0.14	0.050	0.056	2.4	2.5	1.0
		0.076–1.2	0.028–0.078	0.0078–0.10	1.2–7.5	1.0–16	0.54–4.6
36	Poland, Porażyn, 2008, *n* = 15	0.33 ± 0.13	0.071 ± 0.026	0.049 ± 0.029	5.1 ± 2.4	8.8 ± 5.1	1.8 ± 0.9
		0.28	0.070	0.041	5.4	8.4	1.9
		0.17–0.53	0.037–0.11	0.018–0.13	2.1–10	2.3–17	0.32–3.0
37	Poland, Rogalin, 2008 *n* = 4	0.17 ± 0.04	0.058 ± 0.010	0.030 ± 0.017	2.6 ± 2.1	7.2 ± 5.3	5.7 ± 6.0
		0.17	0.064	0.027	2.6	4.9	3.7
		0.12–0.21	0.029–0.077	0.013–0.053	0.27–5.0	4.0–15	0.93–14
38	Poland, Anastarzewo near Konin, 2003 *n* = 17 (85)	0.17 ± 0.04	0.041 ± 0.028	0.020 ± 0.034	5.3 ± 2.6	17 ± 9	4.7 ± 4.1
		0.17	0.035	0.0095	4.6	16	3.5
		0.10–0.23	0.012–0.13	0.0078–0.12	1.0–13	1.8–37	0.41–13

*NA* not analyzed

^ψ^Place (see Fig. 1)

*Number of individuals

^#^number of composite samples

**number of individuals in a pool (in parentheses)

**Table 2:** Mercury (mg kg^-1^ dry matter) in composite samples of whole fruiting bodies or caps of *Suillus luteus* from different localizations in Poland

Region	Place and year	Number of individuals	Fungus	Hg
Pomerania	(4)^ψ^ Darżlubska Wilderness - Ostrowo, 2003	33	Whole	0.21
	(6) Strzebielino, 2006	31	Whole	0.18
	(7) Słupsk region, 2003	40	Whole	0.30
	(10) Stężyca, 2000	38	Whole	0.21
	(12) Trójmiejski Landscape Park - Niedźwiednik, 1995	19	Whole	0.26
	(14) Sobieszewo Island, 1999	29	Whole	0.11
Kociewie land	(15) Zaborski Landscape Park, 1998	17	Whole	0.17
	(17) Tuchola Pinewoods - Pelplin, 2000	24	Whole	0.14
Mazury land	(25) Kętrzyn (outskirts), 2000	96	Whole	0.30
	(26) Giżycko (outskirts), 2000	15	Caps	0.29
	(28) Szczytno (outskirts), 2003	16	Whole	0.21
Warmia land	(27) Olsztynek (outskirts), 2000	48	Whole	0.15
	(29) Puchałowo, 2001	20	Whole	0.12
Kujawy land	(34) Lubraniec, 2000	73	Whole	0.17
	(35) Mursk, Goreń, Kukawy, 2001	45	Whole	0.16

^ψ^Place (see Fig. 1)

All individual fruiting bodies selected for this study were mature and in good body condition (not infected by insects). They were cleaned up from any visible plant vegetation and soil debris with a plastic knife. To get insight into the distribution of Hg between the two major morphological parts of the fruiting bodies of mushrooms, the individual mushrooms from several places were separated into cap (with skin) and stem. Next, the individual cap and stem samples were sliced using a plastic knife and dried separately or in a pool accordingly. Thereafter, for drying, the mushroom samples were placed into plastic basket of the electrically heated commercial dryer for vegetables and dried at 65 °C to constant mass. Dried fungal materials were pulverized in a porcelain mortar and kept in brand new sealed polyethylene bags under dry conditions. The soil samples, free of any visible organisms, small stones, sticks, and leaves were air dried at room temperature for several days under clean conditions and further dried at 65 °C to constant mass. Next, the soil samples were ground in a porcelain mortar, sieved through a pore size of 2-mm plastic sieve, and thereafter, stored in brand new sealed polyethylene bags under dry conditions.

Double-distilled water was used in all preparations. Mercury standard solution of 1.0 mg mL^−1^ Hg was obtained from the 10 mg mL^−1^ standard stock solution. Blank and 100, 150, and 200 μL of 1.0 mg mL^−1^ Hg standard solutions were injected into the analyzer for the construction of a calibration curve, which was prepared new each week.

The determinations of total Hg content of fungal and soils samples was performed using cold-vapor atomic absorption spectroscopy (CV-AAS) by a direct sample thermal decomposition coupled with gold wool trap of Hg and its further desorption and quantitative measurement at wavelength of 253.7 nm. The analytical instrument used was mercury analyzer (MA-2000, Nippon Instruments Corporation, Takatsuki, Japan) equipped with auto sampler and operated, respectively, at low and high mode (Jarzyńska and Falandysz 2011).

Quality of determinations was assured by the analysis of blank samples and one certified fungal reference material CS-M-1 (dried fruit-bodies of mushroom *S. bovinus* (L.) Roussel 1796; Institute of Nuclear Chemistry and Technology, Warsaw, Poland) is 0.174 ± 0.018 Hg mg kg^−1^ dm Hg and our result (*n* = 27) was 0.180 ± 0.008 mg kg^−1^ dm; for CS-M-2 (dried mushroom powder *Agaricus campestris* L.) the declared Hg content is 0.164 ± 0.004 Hg mg kg^−1^ dm, and our result (*n* = 14) was 0.167 ± 0.005 mg kg^−1^ dm and for CS-M-4 (dried mushroom powder *Leccinum scabrum*), the declared content is 0.465 ± 0.024 Hg mg kg^−1^ dm, while present determinations (*n* = 9) showed 0.448 ± 0.015 mg kg^−1^ dm. Two blanks with each set of ten samples of mushroom or soils samples using analyzer type MA-2. The limit of detection (LOD) in our study was 0.003 mg Hg kg^−1^ dm, and the quantification limit (LOQ) was 0.005 mg Hg kg^−1^ dm.

## Results and discussion

### Mercury in fruiting bodies—cap and stem

A summary of the results obtained including the results of Hg concentration in caps and stems of *S. boletus*, Hg the substrate, as well as the values of quotients of element concentrations in cap to stem (Q_C/S_) and the bioconcentration factor, BCF (cap/stem to soil substratum) are given in Table 1. The mean Hg concentrations of the caps of *S. boletus* collected between 2000 and 2013 from Poland as well as individuals collected in 1995 from Umeå region in Sweden as investigated are generally low ranging from 0.070 to 0.69 mg kg^−1^ dry matter (dm) and varied widely from site to sites. The mean Hg in caps was between 0.13 ± 0.05 and 0.33 ± 0.13 mg kg^−1^ dm. A median value of 0.15 mg kg^−1^ dm was observed for samples from Umeå in Sweden, the Seaside Landscape Park, and Parchowo sites in Poland as well as for the pooled samples from Belarus. An elevated Hg in caps at 0.40 mg kg^−1^ dm was also observed for samples from area nearby to Wysokie site.

The stem mean values ranges from 0.038 ± 0.014 mg kg^−1^ dm (Szczecinek site) to 0.095 ± 0.038 mg kg^−1^ dm (Seaside Landscape Park site) with individual values ranging from 0.012 to 0.86 mg kg^−1^ dm. Similar mean values were observed for the stem samples of the Augustów Primeval Forest (0.040 ± 0.012 mg kg^−1^ dm) and stem from Anastarzewo (0.041 ± 0.028 mg kg^−1^ dm). A fluctuation of Hg content both in caps and stipes of *S. luteus* foraged in the northern part of Poland over the years 1995–2010 (Tables 1 and 2) could be observed but with not a consistent tendency. In earlier time-trend study of Hg in *Paxillus involutus* (Batsch) Fr. 1838 and *Macrolepiota procera* (Scop.) Singer 1948, levels were similar for the years surveyed (Brzostowski et al. 2011b; Falandysz and Brzostowski 2007; Falandysz et al. 2007b; Gucia et al. 2012a).

The mean Hg Qc/s values range from 1.8 ± 0.4 to 5.3 ± 2.6 (overall range of 0.27–13) indicating that the caps had more Hg than the stems. Similar Qc/s values of approximately 2 were observed for samples from many of the sites studied including Umeå in Sweden (2.1) and sites in Poland: Darżlubska Wilderness (2.0), Seaside Landscape park (2.0), Parchowo (2.1), Bydgoszcz forests, Białe Błota (2.2), Ciechocinek (1.8) as well as for Wysokie (2.2), Borysow in Belarus (2.1) and Kostryca in Belarus (2.4). This shows that the caps contain at least twice the amount of Hg observed in the stems.

### Hg in soil

Hg in the substrate were mostly low ranging from 0.0030 to 0.15 mg kg^−1^ dm with mean values varying from 0.0078 ± 0.0035 mg kg^−1^ dm (for Szczecinek) to 0.053 ± 0.025 mg kg^−1^ dm (for Włocławek site). The Hg concentrations of this study are generally low though somewhat comparable to results of some earlier studies of Hg in *S. bovinus* and substrate from other parts of Poland (Falandysz and Kryszewski 1996; Falandysz and Chwir 1997). A high level of Hg in soil of 0.67 mg kg^−1^ dm was observed for specimens from Kaszuny, while reason is unknown. Comparable mean Hg in soil values were also obtained for Sweden (0.048 ± 0.041 mg kg^−1^ dm) and the Porażyn (0.049 ± 0.029 mg kg^−1^ dm) sites. The sites surveyed have no known historic or present Hg emissions and are without geochemical Hg anomalies. Deposition of airborne Hg because of emissions from the anthropogenic sources and long-range transport and in part, also natural sources of emissions (volcanoes, volatilization from lithosphere, water, and biotic-based surfaces) could contribute to accumulation and retention of Hg in topsoil of forests in Poland, while typical levels observed in top 0–10 and 0–15 cm layer are below 0.05 mg kg^−1^ dm (Falandysz 2002, 2014; Falandysz et al. 1996, 2015).

### Bioconcentration factor

To estimate the potential of mushrooms and other biota to bioconcentrate chemical elements/compounds from substratum, it has become traditional to calculate the bioconcentration factor (BCF; also known as transfer factor, TF or enrichment factor, EF)—concentration quotients of the element/compound in fruiting body (cap or stem)—to that of the substrate underlying the fruiting body. The values of the bioconcentration factor provides information on whether the element is actively absorbed and accumulated (BCF > 1), or not, i.e., excluded (BCF < 1). The BCF values showed that *S. bovinus* mushroom has the potential to accumulate mercury. Mean Hg BCF values varied between 3.6 ± 1.3 (for Augustów Primeval Forest site) 42 ± 18 (Szczecinek site) for caps (individual values ranging from 1.0 to 75) and from 1.2 ± 0.4 (for Augustów Primeval Forest site) to 8.0 ± 2.0 (Ciechocinek site) for stem (individual values ranging from 1.2 to 20). Bioaccumulation of Hg by the *S. luteus* mushroom was observed for virtually all the sites for both the caps and the stems though much more for the caps. The median BCF of caps from Sweden is about one eighth that for the Szczecinek site. The mean BCF values for caps were generally high with all of the sites having mean BCF >1 (same for the stems). The median BCF value for the Szczecinek site was 39 and mean ± SD was at 42 ± 18, and what is about eight times higher for less polluted soil substratum (median at 0.0072 mg kg^−1^ dm), when compared to that for the samples from Sweden with median BCF at 5.1 and mean ± SD at 4.6 ± 2.0 for more polluted soil (median at 0.029 mg kg^−1^ dm) (Table 1). Compared to some other mushroom species and sites, the *S. luteus* mushroom is not so good as a bioaccumulator of Hg. For instance, studies of the carpophores of Puffball (*Lycoperdon perlatum* Pers 1796) reported BCF of up to 110 (Falandysz et al. 2001) and 35 ± 23–100 ± 100 and 200 ± 91 (Falandysz et al. 2002, 2012), and 420 (Falandysz et al. 2003) for samples from Poland.

### Estimating intakes

The occurrence of essential and toxic trace elements in edible mushrooms collected in the wild as well as their intake rates, nutritional benefits, and likely risks are of primarily concern to consumers. Consequently, researches have been focusing on providing such information. In estimating likely risks from Hg intakes on consumption of caps and stems of *S. luteus*, the reference dose (RfD; 0.0003 mg kg^−1^ body mass daily) and Provisionally Tolerable Weekly Intake (PTWI) value (PTWI of 0.004 mg kg^−1^ bm) were used as reference values (JECFA 2010; US EPA 1987).

The median Hg in caps and stems of *S. luteus* (dry weight) varied from 0.11 to 0.28 and 0.035 to 0.097, respectively. These dry weight values amount to Hg content of 0.011 to 0.028 mg kg^−1^ dm and 0.0035 to 0.0097 mg kg^−1^ dm fresh weight, respectively, assuming moisture content at 90 %. Mercury contained in fruiting bodies of mushrooms is hardly to be removed during their boiling (blanching) (Falandysz and Drewnowska 2015), which is a mandatory procedure as well as removing of skin from the cap before further processing of *S. luteus* for soup or frying, because of high content of mucus composed of polysaccharides, which can lead to dehydration of consumer body. The estimated Hg intake by a 70-kg person on the consumption of 300-g cap sample (stems of this mushroom are usually cutoff and not collected) ranges from 0.0033 to 0.0084 mg (from 0.000047 to 0.00012 mg kg^−1^ body mass) considering the median Hg values mentioned earlier. Considering the established PTWI Hg intake limits, the consumption of fresh caps of *S. luteus* mushrooms at a rate of 300 g week^−1^ especially during the mushrooming season, would not result in Hg intakes that exceeds the PTWI limit.

## Conclusion

The mercury contents of *S. luteus* mushrooms from 38 sites as well as the substrate soils were determined in this study so as to evaluate the ability of *S. luteus* to bioconcentration Hg and probable dietary intake of element and risk. The results obtained showed the Hg content of *S. luteus* to vary from 0.13 ± 0.05 to 0.33 ± 0.13 mg kg^−1^ dm for caps and from 0.038 ± 0.014 to 0.095 ± 0.038 mg kg^−1^ dm in stems. *Suillus luteus* moderately bioaccumulate Hg and the mean values of the bioconcentration factor (BCF) ranged from 3.6 ± 1.3 to 42 ± 18. The consumption of *S. luteus* poses no toxicological concerns as the estimated intake levels from *S. luteus* mushroom collected from unpolluted forests among the investigated site did not exceed the established limits of Hg intake from foods such as the RfD and the PTWI.
